# Nano-Characterization, Composition Analysis, and Anti-Inflammatory Activity of American-Ginseng-Derived Vesicle-like Nanoparticles

**DOI:** 10.3390/molecules29153443

**Published:** 2024-07-23

**Authors:** Taiping Li, Huan Wang, Wenjie Bi, Yonghui Su, Yongai Xiong, Songsong Wang, Liwen Han

**Affiliations:** 1 School of Pharmaceutical Sciences & Institute of Materia Medica, Shandong First Medical University & Shandong Academy of Medical Sciences, Jinan 250017, China; 2 Key Laboratory of Basic Pharmacology of Ministry of Education, Zunyi Medical University, Zunyi 563006, China; 3 James Graham Brown Cancer Center, University of Louisville, Louisville, KY 40292, USA; 4 State Key Laboratory for Quality Ensurance and Sustainable Use of Dao-di Herbs, Beijng 100700, China

**Keywords:** American ginseng, nano-characteristics, ginsenosides, zebrafish, anti-inflammatory

## Abstract

Medicinal plant-derived vesicle-like nanoparticles can carry chemical components and exert intercellular activity due to the encapsulation of nanostructures. American ginseng is well known as a traditional herb and is commonly used in clinical decoctions. However, the nano-characteristics and chemical composition of American-ginseng-derived vesicle-like nanoparticles (AGVNs) in decoctions are unclear. In this study, the gradient centrifugation method was used to extract and isolate AGVNs. A metabolomic method based on high-resolution mass spectrometry was established to analyze small molecules loaded in AGVNs. Zebrafish and RAW264.7 cells were employed to investigate the anti-inflammatory effects of AGVNs. The results showed that the particle size of AGVNs was generally 243.6 nm, and the zeta potential was −14.5 mV. AGVNs were found to contain 26 ginsenosides (14 protopanaxadiols, 11 protopanaxatriols, and 1 oleanolic acid). Ginsenoside Rb1 and malonyl-ginsenoside Rb1 tended to be enriched in AGVNs. Moreover, AGVNs were found to exert anti-inflammatory effects by reducing macrophage migration in zebrafish and regulating inflammatory factor (NO, TNF-α, IL-6, IL-10) secretion in RAW 264.7 cells. The characterization and analysis of AGVNs provide references and data that support the development of nanoscale anti-inflammatory substances from medicinal plants.

## 1. Introduction

Medicinal plant-derived vesicle-like nanoparticles (VNs, 50–1000 nm) are a new type of micro nanoparticles found in fresh tissues or decoctions of lots of medicinal plants [1,2]. More and more researchers have reported that VNs display distinct bioactivities different from conventional small molecules [3,4,5]. Chemical composition is regarded as the main cause of the diverse functions of these VNs. In the clinical application of traditional medicine in Asian countries, medicinal plants or herbs are more often used to treat patients with their decoction rather than fresh plants. At present, a large number of studies have focused on VNs obtained from fresh tissues, but the characteristics of the composition of those in the decoction have not been thoroughly investigated.

American ginseng (AG) is a well-known medicinal plant that is used as a herbal medicine for the treatment of cardiovascular and cerebrovascular diseases and has anti-cancer, anti-inflammatory, and anti-aging effects due to its nourishing properties [6,7]. This plant is rich in ginsenosides (protopanaxadiol (PPD), protopanaxatriol (PPT), and oleanolic acid (OA)). Published research has shown that the VNs of ginseng carry ginsenoside Rg3 and exert anti-inflammatory effects by regulating macrophage polarization [8]. In addition, they can fight gliomas through the blood-brain barrier [9]. AG is a medicinal and edible homologous plant in the genus Panax ginseng; however, there is little information on AG-derived VNs (AGVNs), and there are no relevant characterization analyses or activity reports.

In this study, an analytical workflow was established to focus on AGVNs, reveal their nanostructure, investigate loaded ginsenosides, and examine the anti-inflammatory activities of AGVNs. These results will contribute to a better understanding of the structural and biological characteristics of AGVNs, a new nanoparticle from AG.

## 2. Results

### 2.1. Surface Characterization of AGVNs

**Figure 1:**
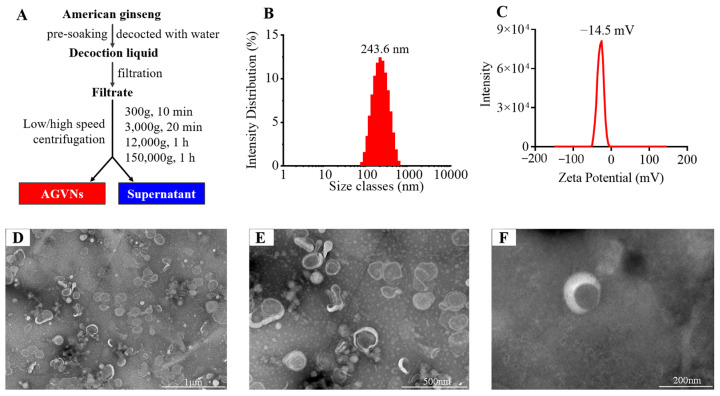
Extraction process and general characterization of AGVNs. (**A**) Extraction steps for AGVNs. (**B**) Zeta potentiometric results. (**C**) Nanoparticle size determination results. (**D**–**F**) Transmission electron microscopy results for AGVNs at different fields of view (1 μm, 500 nm, and 200 nm).

In this work, 20 g of AG was extracted and concentrated using differential centrifugation to provide 350 mL of concentrate. After differential centrifugation, 1 mL of PBS was used to dissolve the AGVNs (Figure 1A). The typical teacup-like structure of AGVNs (Figure 1D–F) was observed at different magnifications through transmission electron microscopy with a particle size concentrated at 243.6 nm (Figure 1B), a stable zeta potential of −14.5 mV (Figure 1C), and a total protein concentration of 515.87 ug/mL.

### 2.2. Validation of the Method of Composition Analysis

**Figure 2:**
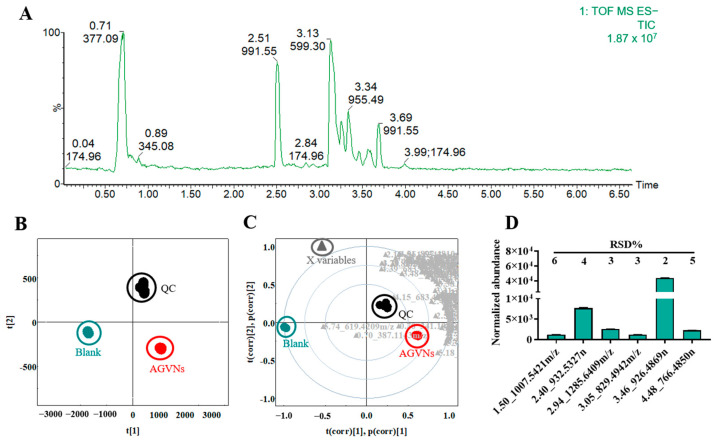
Total ion chromatogram (TIC) and multivariate statistics of the AGVNs. (**A**) TIC collection results for the ESI^−^ from the AGVNs. (**B**) Results of the principal component analysis of the ESI^−^ in multiple groups. (**C**) Multiple groups in the Loading Bi plot of the ESI^−^. (**D**) The normalized plot of the ion peak area in the QC.

First, the stability of the sample processing method was analyzed. The total ion chromatogram of AGVNs was collected in this study (Figure 2A). The ion flow was mainly at 1–4 min (mobile-phase gradient (A) 35–56%); as can be seen from previous reports, this was the mobile-phase composition of the main peak of ginsenosides [10,11]. Each group showed good clustering in a multivariate statistical analysis (PCA, Hotelling T2 Ellipse (95%) = 3301; 858, R2X [1] = 0.9195, R2X [2] = 0.0621); QC reflected the ion information of all samples and was located near the coordinate origin (Figure 2B), and according to the Loading Bi plot, the |pCorr| of X variables mainly converged at 0.5–1 (Figure 2C), contributing significantly to the difference between groups. Finally, we randomly extracted the peak areas of the ions from the six QC samples (Figure 2D), and their RSD% range was 2–6. Therefore, we estimated the stability of the sample processing method and the reliability of the statistical model.

### 2.3. Characterization of the Information on Ginsenosides in AGVNs

Ginsenosides, which contain polyphenolic hydroxyl groups and some carboxyl functional groups, have a higher ionization efficiency at the ESI^−^. The aqueous solutions of the AGVNs were quickly separated within 6.5 min with the UPLC method developed in this research, and typical fragments with sugars as the primary detachment group were detected.

#### 2.3.1. Ginsenosides of PPD

**Figure 3:**
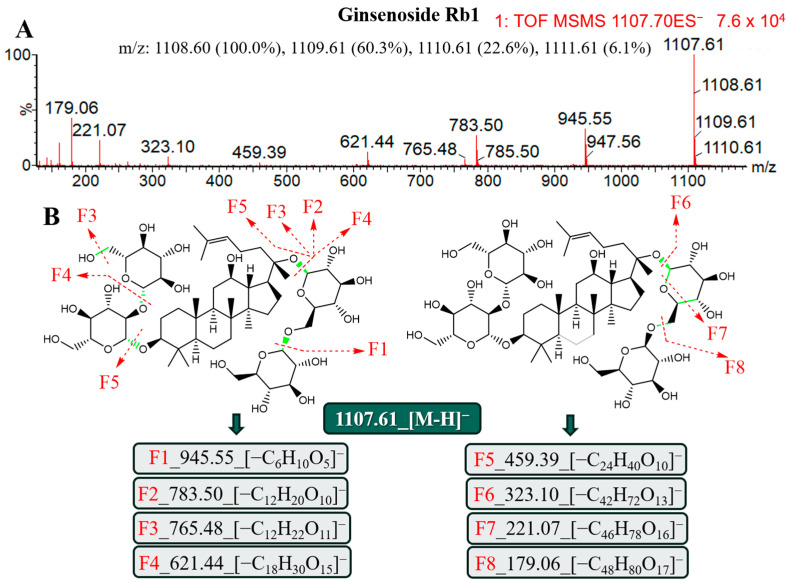
Mass spectrometry characteristics and lysis rule analysis of the ginsenoside Rb1 under the ESI^−^. (**A**) Secondary mass spectrometry information of the ginsenoside Rb1. (**B**) Molecular structure and the possible lysis pathway of the ginsenoside Rb1.

PPD is a tetracyclic triterpene saponin of the dammarane type, and its saponin is 20(S)-protoginselenediol with no substitution at position C6 [12]. In the Chinese Pharmacopoeia, ginsenoside Rb1 acts as an index for assessing the quality of AG. A high intensity of 1108.6016n was seen at 3.13 min during MS scanning, and the database matched it with ginsenoside Rb1. To validate this, 3.13_1108.6016n was used to conduct MS/MS scanning at high energy, and the results are displayed in Figure 3. At the same time, 1107.61*m*/*z*_[M-H]^−^ and an array of desugared and dehydrated ions were identified, including 945.55 *m*/*z* (F1), which was produced by the ginsenoside Rb1 after removing glucose under the ESI^−^, and 783.50 *m*/*z* (F2), which was created by removing two molecules of glucose; we also observed the ions F4_621.44*m*/*z* and F5_459.39*m*/*z*, which had three and four molecules of glucose removed, respectively. Additionally, the shed glucose also produced a clear MS/MS peak (F8_179.06*m*/*z*), indicating that the system’s MS/MS profile was formed at the proper collision energy (35–45 V), which was consistent with the report mentioned above and occurred at precisely the same time as the peak of standard for the ginsenoside Rb1 [10].

#### 2.3.2. Ginsenosides of PPT

**Figure 4:**
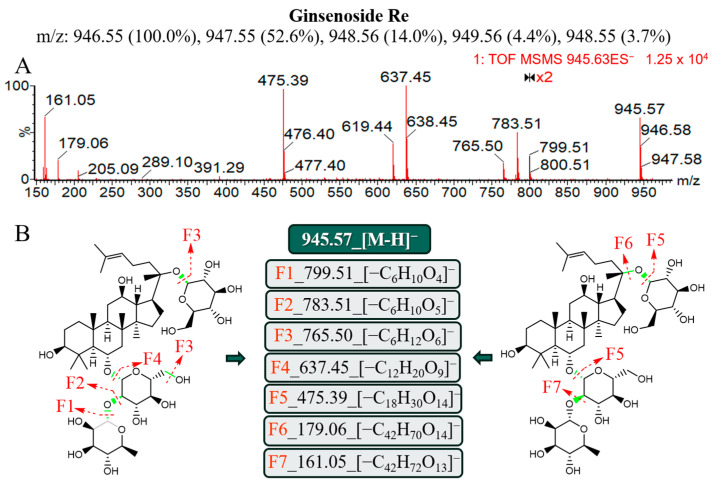
Mass spectrometry characteristics and lysis rule analysis of the ginsenoside Re under the ESI^−^. (**A**) Secondary mass spectrometry information of the ginsenoside Re. (**B**) Molecular structure and the possible lysis pathway of the ginsenoside Re.

In contrast to the PPD-type ginsenoside, PPT is part of the same class of tetracyclic triterpene saponins as dammarane; its saponin is 20 (S)-protoginestriol, and its parent nucleus’ C6 contains hydroxyl groups [12]. Similarly, the Chinese Pharmacopoeia uses the ginsenoside Re as an index for assessing the quality of AG. The known compound library was compared with the primary MS data, and it was discovered that 2.50_945.5414*m*/*z* may be ginsenosides (Re_[M-H]^−^). To confirm this, we searched the literature using standards and compared multiple characteristic fragments in MS/MS (945 *m*/*z*, 783 *m*/*z*, 637 *m*/*z*, 475 *m*/*z*) [10,11], and the MS/MS results in this study were found to be highly consistent with previously reported and other fragment ions that were found at the same time (Figure 4A). It can be seen that the 6-position hydroxyl group of the ginsenoside Re was replaced by glucose–rhamnose, while the F1 fragment was 799.51 *m*/*z* and was formed by derhamnose. F4 (637.45 *m*/*z*) was the characteristic ion formed by the simultaneous shedding of disaccharides (glucose–rhamnose); 475.39 *m*/*z* was the ginsenoside Re when ESI^−^ dropped three glycosyls at the same time to generate ions (two molecules of glucose and one molecule of rhamnose), and independent glycan ions (179.06 *m*/*z* (glucose) and 161.05 *m*/*z* (rhamnose)) also had strong signals (Figure 4B).

#### 2.3.3. Ginsenosides of OA

**Figure 5:**
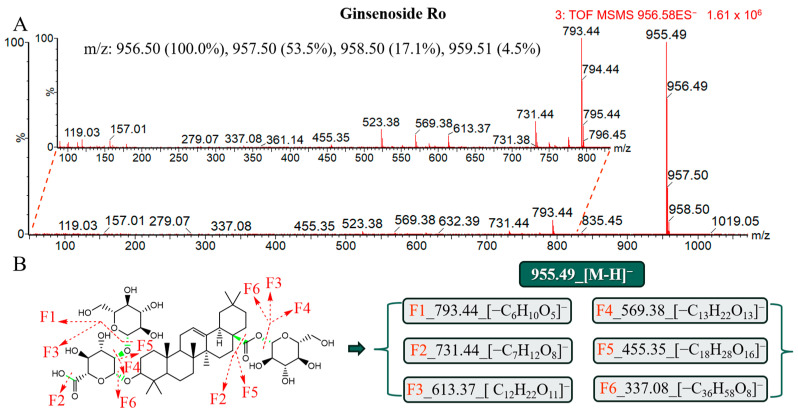
Mass spectrometry characteristics and lysis rule analysis of the ginsenoside Ro under the ESI^−^. (**A**) Secondary mass spectrometry information of the ginsenoside Ro. (**B**) Molecular structure and the possible lysis pathway of the ginsenoside Ro.

Aldonolic pentacyclic triterpenoid derivatives include ginsenosides of the OA class. OA, a representative component of the ginsenoside Ro, contains a hydroxyl group at the C3 position and a carboxyl group at the C28 position that serve as attachment sites for functional groups. It was found by analyzing the MS/MS results (Figure 5) that 3.34_956.4973n produced an excimer ion peak of 955.49*m*/*z*_[M-H]^−^ in the ESI^−^, 793.44 *m*/*z* was the ion (F1) formed after desugaring, and another characteristic peak of 569.38 *m*/*z* was a molecular ion peak that directly broke at the C28 position and continued to drop glucose to form F4. F5 was another key MS/MS peak, which may have been after carboxyl terminal desugaring. The 455.35 *m*/*z* formed after disaccharide was shed at the C3 position, and sure enough, after standard alignment, the peak at 3.34 min was determined to be ginsenoside Ro.

**Figure 6:**
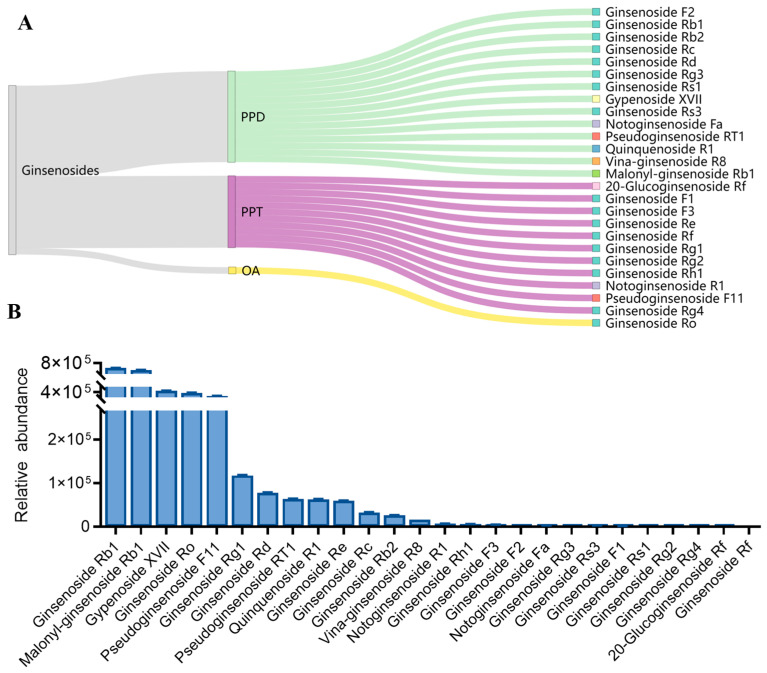
Visual analysis of the categories and relative content of ginsenosides in AGVNs. (**A**) Sanji diagram of the ginsenoside categories. (**B**) Comparison of the intensities of the 26 ginsenosides in the AGVNs.

There were ginsenosides in the AGVNs, including 11 PPT, 14 PPD, and 1 OA (Figure 6A), as determined through the examination of UPLC-Q/TOF-MS/MS. Table S1 lists the detailed experimental data. As shown in Figure 6B, the top ten ginsenosides in AGVNs from high to low were ginsenoside Rb1, malonyl-ginsenoside Rb1, gypenoside XVII, ginsenoside Ro, pseudoginsenoside F11, ginsenoside Rg1, ginsenoside Rd, pseudoginsenoside RT1, quinquenoside R1, and ginsenoside Re.

### 2.4. Anti-Inflammatory Phenotypic Assessment of AGVNs In Vivo

**Figure 7:**
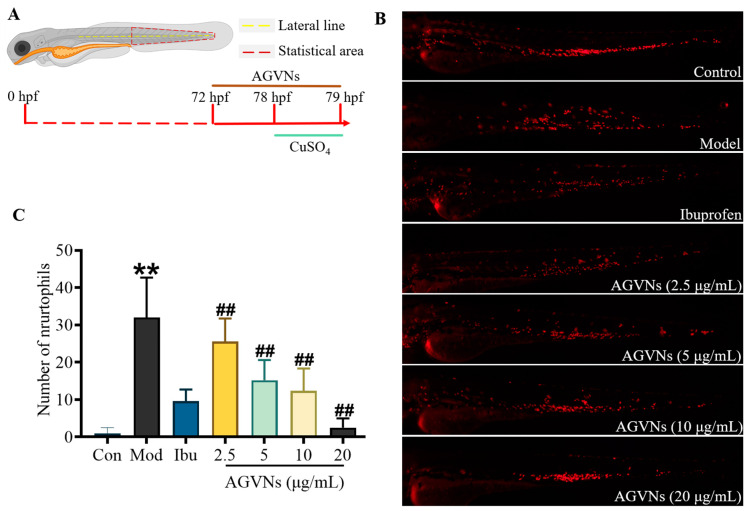
Evaluation of the effect of AGVNs on CuSO_4_-induced inflammatory models. (**A**) Schematic of model induction and AGVNs intervention. (**B**) Representative red-fluorescent neutrophils from different groups (bar = 200 μm). (**C**) Quantitative analysis of the number of neutrophils in the zebrafish inflammatory models before and after the administration of AGVNs. The red dashed quadrilateral box represents the quantitative area. Data are expressed as the mean ± SEM; ** *p* < 0.01 vs. the control group; ^##^ *p* < 0.01 vs. the model group (CuSO_4_).

CuSO_4_ has the advantage of being noninvasive to zebrafish models, with macrophages migrating near lateral-line mounds after inflammation occurs [13]. At 3 days post-fertilization, the primary lateral-line nervous system of zebrafish was established. As shown in Figure 7A, Cu^2+^ was able to quickly damage lateral-line hair cells, induce oxidative stress, and cause macrophage and granulocyte infiltration [14]. The number of neutrophils in the CuSO_4_ group was also significantly higher than that in the control (*p* < 0.05). Ibuprofen was able to exert significant anti-inflammatory effects and reduce the number of inflammatory cells, while CuSO_4_ significantly increased the number of neutrophils and caused inflammation. Zebrafish inflammatory models also significantly decreased the number of inflammatory cells after the administration of AGVNs, indicating their anti-inflammatory biological activity (Figure 7B,C).

### 2.5. In Vitro Uptake and Anti-Inflammatory Evaluation of AGVNs

**Figure 8:**
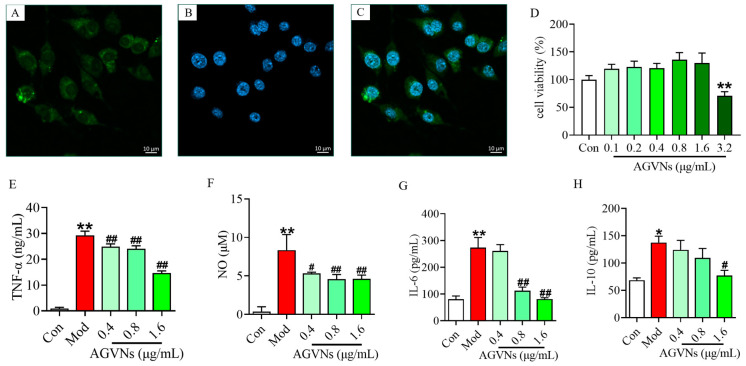
Analysis of the role of macrophage uptake of AGVNs and the regulation of inflammatory factors by AGVNs. (**A**–**C**) The results of DiO staining, DAPI staining, and merge staining under confocal microscopy. (**D**–**H**) Quantitative results of AGVNs in terms of macrophage secretion of TNF-α, NO, IL-6, and IL-10. Data are expressed as the mean ± SEM; * *p* < 0.05, ** *p* < 0.01, vs. the control group (DMSO); ^#^ *p* < 0.05, ^##^ *p* < 0.01, vs. the model group (LPS).

DiO is a lipophilic fluorescent dye that can be used to stain cell membranes and other fat-soluble biological structures, and upon entering the cell membrane, DiO diffuses throughout the cell membrane and displays a green color (Figure 8A). A blue color of LPS-induced RAW264.7 was observed with DAPI reagents (Figure 8B), while an uptake interaction phenomenon was observed under a confocal microscope after the incubation of AGVNs and RAW264.7 for 24 h (Figure 8C). The BCA concentration of AGVNs at 0.1–1.6 μg/mL had no significant effect on the activity of RAW264.7 cells (Figure 8D). Importantly, AGVNs (0.4, 0.8, 1.6 μg/mL) significantly reduced the TNF-α and NO levels (Figure 8E,F), AGVNs (0.8, 1.6 μg/mL), significantly inhibited IL-6 secretion (Figure 8G), AGVNs (1.6 μg/mL), and significantly reduced the IL-10 levels (Figure 8H); as shown by the zebrafish analysis, AGVNs exhibited significant anti-inflammatory effects in vivo and in vitro (*p* < 0.05).

## 3. Discussion

Natural products have gradually become a source of substances for the maintenance of human health. Scientists have identified many beneficial compounds and combinations from animals, plants, and microorganisms. VNs are one of the new forms; they were originally extracted from biological samples from animals, and in the last decade, more and more research has been carried out on plant-derived VNs, especially in terms of their internal biological activity, loading properties, bioavailability, and delivery potential. Compared with animal-derived VNs, plant-derived VNs are more widely available because they are contained in fruits and vegetables; they minimize the risk of zoonotic diseases, and the cost of batch preparation is also lower [15,16]. Due to their significant biological activity, VNs from medicinal plants have more potential for development than those from fruits and vegetables.

There are many methods for the extraction of VNs, such as differential centrifugation and sucrose gradient centrifugation, but the extraction efficiency is difficult to improve, leading to the in vitro analysis of cells [17]. In this study, AGVNs were identified in a decoction using differential centrifugation. We took advantage of an integrated zebrafish model, the small sample size required, and the short developmental cycle allowed us to study AGVNs, which allowed for rapid evaluation of their activity and the ability to same them, making up for the need for more AGVNs when using mice. In addition, the Tg (lyz: DsRED2) line of zebrafish used in the in vivo study was a transgenic line that allowed the fluorescent detection of the number of neutrophils, which was advantageous for the evaluation of the anti-inflammatory effect of AGVNs. In vivo, the anti-inflammatory effect of AGVNs (BCA protein concentration of 20 μg/mL) was similar to that of the positive control group (Ibu), which indicates the potential to develop nanoscale anti-inflammatory substances.

VNs are loaded with both large and small molecules. Ginsenosides are the most well-known active substances in AG [18]. Water and alcohol can be used to extract saponins from plant tissues; however, the presence of saponins in VNs and whether there is a difference in the saponin composition with AG are unknown. Therefore, we used the metabolomic method of UPLC-Q/TOF-MS to identify ginsenosides in AGVNs. This study found that AGVNs had the highest levels of ginsenoside Rb1 and malonyl-ginsenoside Rb1. This provides a reference for the analysis of the small-molecule composition of AGVNs.

The inflammatory response is an important link in the disease process and is usually treated with steroid drugs and immunosuppressants [19]. However, similarly to cancer treatments, the efficacy of these anti-inflammatory therapies is severely limited due to their non-specific targeting and off-target toxicity, and there is a risk of immune and gastrointestinal reactions [20]. The development of new substances from natural products is the direction of scientific research; due to the wide variety of existing fruits, vegetables, and medicinal plants that have been reported to be anti-inflammatory, their VNs also have more worthy functions. For example, in vitro and in vivo studies have shown that mammalian VNs and probiotic VNs derived from mesenchymal stem cells have anti-inflammatory properties and can be used to treat inflammatory diseases, such as arthritis and chronic kidney disease [21,22]. Grapefruit VNs may be able to enhance the anti-inflammatory capacity of intestinal macrophages and maintain the expression of E-cadherin in intestinal epithelial cells, thereby conferring a protective effect in vivo [23]. In this study, AGVNs were observed to be similar to the previous phenomenon of ginger VNs being taken up by cells [24], they had a significant regulatory effect on the inflammatory factors (TNF-α, NO, IL-6, and IL-10) secreted by RAW246.7 cells induced by LPS, and they exhibited anti-inflammatory effects in vitro.

In summary, AGVNs were found in the aqueous extract of AG, and their appearance, particle size, and chemical composition were reported in detail; it was found that AGVNs could be taken up by macrophages and exerted anti-inflammatory effects. These results provide important data for an in-depth understanding of the structure and biological activity of AGVNs, and they are conducive to promoting the discovery of more nanostructured active substances from traditional medicinal plants.

## 4. Materials and Methods

### 4.1. Chemicals and Reagents

AG was purchased from Tongrentang Pharmacy (Jinan, China) and was identified by Liwen Han of the Department of Pharmacy of Shandong First Medical University. Ultrapure water was purchased from Watsons Food and Beverage Co., Ltd. (Guangzhou, China). Phosphate-buffered saline (PBS) and Dulbecco’s modified Eagle’s medium (DMEM) were acquired from Solarbio Biochemical Co., Ltd. (Beijing, China). In addition, 2-(4-Amidinophenyl)-6-indolecarbamidine dihydrochloride (DAPI), 3,3′-dioctadecyloxacarbocyanine perchlorate (DiO), and BCA kits were purchased from Beyotime Biotechnology Technology Co., Ltd. (Shanghai, China). Acetonitrile (HPLC grade) and methanol (HPLC grade) were purchased from Macklin Biochemical Co., Ltd. (Shanghai, China). Leucine enkephalin, sodium formate, and dimethyl sulfoxide were acquired from Aladdin Biochemical Technology Co., Ltd. (Shanghai, China). Ginsenosides (Rb1, Rg1, Ro, and Re) were purchased from Yuanye Biochemical Technology Co., Ltd. (Shanghai, China). Murine RAW264.7 cells were acquired from Procell Biochemical Technology Co., Ltd. (Wuhan, China). An inflammatory factor detection kit [tumor necrosis factor α (TNF-α), interleukin 6 (IL-6), interleukin 10 (IL-10), and nitric oxide (NO)] was purchased from Jiancheng Biotechnology Co., Ltd. (Nanjing, China).

### 4.2. Purification and Characterization of AGVNs

For AG (20 g), 200 mL of ultrapure water was added to soak for 30 min, boiled, decocted for 1 h (twice), and filtered to purify the decoction liquid; the filtrate was concentrated with an auto-evaporator (55 °C), and about 100 mL of decoction liquid was obtained after concentration. Centrifugation was continued for 10 min (4 °C, 300× *g*), the supernatant was centrifuged for 20 min (4 °C, 3000× *g*), and the supernatant was taken for the third time at 4 °C; this was followed by centrifugation at 12,000× *g* for 1 h, and the supernatant was filtered with a 0.45 μm filter membrane. The filtrate was ultracentrifuged for 1 h (4 °C, 150,000× *g*), the supernatant was discarded, the pellet was resuspended in 1 mL of PBS buffer solution (AGVNs), and the AGVNs were broken (4 °C, 1 min) using ultrasonic cell disruption (MIULAB, Hangzhou, China) and stored at −80 °C.

The particle size and zeta potential formed the general characterization of the AGVNs, and this characterization was carried out with a dynamic-light-scattering-based Zeta Sizer Nano ZS Zen3600 (Malvern, UK) (parameter settings: RI: 1.590; absorption: 0.010; dispersant: H_2_O; temperature: 25.0 °C; viscosity: 0.8872 CP). A suitable quantity of AGVNs was deposited in formvar-coated copper grids, allowed to dry at room temperature, and then viewed at 200 kV with a magnification of 38,000× by utilizing a Hitachi H-7650 scanning electron microscope (Hitachi, Japan). Additionally, a BCA protein assay kit was used to measure the amount of protein present in the AGVNs, as directed by the manufacturer. The AGVNs concentration was indirectly determined using the bicinchoninic acid (BCA) method.

### 4.3. Analytical Conditions for Chromatography

The ACQUITY UPLC^®^ system (Waters, Milford, MA, USA), consisting of a quaternary solvent manager and a sample manager, was equipped with an HSS T3 column (2.1 mm × 100 mm id, 1.8 µm; Waters, MA, USA) for a separate mixture of ginsenosides from the AGVNs. The column temperature was 35 °C, and the sample manager temperature was 10 °C. In the gradient elution, solvent A (0.1% FA-H_2_O) and solvent B (ACN) comprised the gradient mobile phase. The optimal gradient elution state was as follows: 0–2 min, 20–50% A; 2–5 min, 50–60% A; 5–6 min, 60–90% A; 6.0–6.5 min, 90–20% A. The flow rate was 0.3 mL.min^−1^, and the injection volume was 2 μL.

### 4.4. Analysis Conditions for Mass Spectrometry

A SYNAPT-XS mass spectrometer (Waters, MA, USA) was equipped with an electrospray ion source to detect sample flow from ultra-performance liquid chromatography at a source temperature of 100 °C. The capillary voltage was 2.5 kV in negative ion mode (ESI^−^). The cone voltage was 40 V and the gas flow rate was 50 L.h^−1^. The nitrogen desolvation flow rate was 800 L.h^−1,^ and the temperature was 450 °C. Ion information for *m*/*z* 50–1500 Da was collected in a data-independent manner, and the cross-collision energy range was 15–45 V. Leucine enkephalins with a concentration of 1 ng/μL and a flow rate of 10 μL.min^−1^ in the ESI^−^ ([M-H] = 554.2615) were used as a reference to obtain an accurate mass. Leucine enkephalins were further utilized to set the mass spectrometer’s detector voltage, sodium formate was applied to optimize the mass axis, and quality control samples created by combining all samples were used to investigate analytical situations and instrument stability to obtain data with a lower level of error.

### 4.5. Metabolomic Data Processing

PPD, PPT, and OA ginsenosides, which are steroid-based and contain polyhydroxyl and carboxyl groups, are the primary components of AG. These chemicals are typically extracted with water and ionized in the ESI^−^. In this work, after filtration through a 0.22 μm membrane, AGVNs were used to extract ginsenosides with quadruple volumes of water. After centrifugation, 2 μL of the supernatant was injected into an ultra-performance liquid chromatography quadrupole time-of-flight coupled with mass spectrometry (UPLC-Q/TOF-MS).

Raw data were preprocessed (peak extraction, peak alignment, and normalization) with the Progenesis QI 3.0 software (Waters, MA, USA) to obtain a matrix containing *m*/*z*, retention time (Rt), and ionic intensity, which were imported into the EZinfo 3.0 software (Waters, MA, USA) for multivariate statistical analysis to screen for differential ions. Principal component analysis (PCA) was used to determine the difference between the AGVNs and a blank (water), and orthogonal partial least squares discriminant analysis (OPLS-DA) was used to calculate the variable importance in the projection value (VIP value) with the statistically normalized abundance of different ions. For the database, the name-*m*/*z* database of ginsenosides was built, and their information was obtained by searching Pubchem (https://pubchem.ncbi.nlm.nih.gov/, accessed on 16 October 2023) and ChemSpider (http://www.chemspider.com/, accessed on 17 October 2023); the above ion matrix was finally initially matched with the database before subsequent characterization and identification. We adopted background eradication, and the data matrix was further screened to exclude ion information with high responses in the blank, quality control (QC) samples, with further screening only for ions with a peak in AGVNs. First, we used the solvent as a blank to remove interference. The ions with VIP > 0 were then subjected to intensity statistics (*p* < 0.05), and the screened ions were matched with the aforementioned database using the Rt and *m*/*z* limit (ΔRt = 0.1 min, Δ*m*/*z* = 5 ppm) and comparisons with standards and the secondary mass spectrometry (MS/MS) reported in the literature were made to confirm the ginsenosides contained in the AGVNs.

### 4.6. Rapid Evaluation of the Anti-Inflammatory Effects of AGVNs

Wild-type AB and transgenic Tg (lyz: DsRED2, in vivo fluorescently labeled neutrophils) zebrafish were supplied by the Zebrafish Research Center of Shandong First Medical University and raised under a 14 h light/12h dark cycle in a commercial zebrafish culture system (Shanghai Haisheng Biological Experimental Equipment Co., Ltd., Shanghai, China). Before the experiment, male and female adult zebrafish at a 2:2 ratio were placed in a mating tank to reproduce the embryos. Zebrafish at 72 h post-fertilization were selected and seeded in six-well plates. Each well contained 5 mL of culture medium with 20 animals and was maintained at 28.5 °C. The control group contained only 0.5% dimethyl sulfoxide, and the BCA concentrations of AGVNs were 2.5 μg/mL, 5 μg/mL, 10 μg/mL, and 20 μg/mL; the concentration of ibuprofen (positive control group) was 4 μg/mL, and the dimethyl sulfoxide limit of all groups was 0.5%. All groups of zebrafish were pre-protected for 6 h, and CuSO_4_ was added to the modeled zebrafish to make the final concentration of 4 mg/L for 1 h [25].

### 4.7. Cell Culture and Viability Assay

Murine RAW264.7 cells were cultured in DMEM containing 10% FBS at 5% CO_2_ and 37 °C, and the medium was changed every 48 h. RAW264.7 cells at 5 × 10^5^/well were placed in 96-well plates and treated with AGVNs (0, 0.1, 0.2, 0.4, 0.8, 1.6, 3.2 μg/mL) for 24 h, and the viability of the cells was detected with CCK-8 according to the manufacturer’s protocol; the incubation time of CCK-8 was 1 h, and the OD value of each well was measured using a microplate reader at 450 nm.

### 4.8. DiO-Labeled AGVNs

DMSO was used to formulate a 10 mmol· L^−1^ DiO solution, 1 mL of AGVNs (1.6 μg/mL) was taken, and 2.5 μL of DiO solution was added; this was stained for 20 min, centrifuged at 150,000× *g* for 1 h, followed by resuspension in PBS for 1 h (150,000× *g*), and the stained solution was washed. RAW264.7 cells were seeded in a dish at 1 × 10^5^ cells/mL, cultured at 37 °C and 5% CO_2_ for 24 h, given AGVNs (1.6 μg/mL), and incubated together for 24 h; the medium was discarded and cells were washed with PBS; 4% tissue fixative solution of 500 μL of PBS was used to fix cells for 15 min; this was removed with a fixative solution and washed with PBS. PBS was added again and shaken for 5 min to remove PBS, and 400 μL of DAPI solution was added to each well for 10 min. The DAPI stain was removed, and cell uptake was observed by confocal microscopy (ZEISS LSM980, Oberkochen, Germany) after washing with PBS.

### 4.9. ELISA

RAW264.7 cells were seeded at 1 × 10^5^ cells/well in a dish and pretreated with AGVNs (0, 0.1, 0.2, 0.4, 0.8, 1.6 μg/mL) for 12 h. Cells were treated with 1 μg/mL LPS for 12 h [26], the cell supernatant was collected after treatment, centrifugation was performed at 10,000× *g* at 4 °C for 5 min, and the detection of the OD values (450 nm) of NO, IL-10, IL-6, and TNF-α was performed according to the manuals of the corresponding kits.

### 4.10. Statistical Analysis

GraphPad Prism 9.0 (GraphPad Software, Boston, MA, USA) was used for quantitative statistics, and all data are represented as the mean ± standard error (SEM). Student’s *t*-test and ANOVA were used to analyze the differences between two or more groups, respectively, and *p* < 0.05 was considered to indicate a statistical difference (* *p* < 0.05, ** *p* < 0.01).

## 5. Conclusions

In conclusion, this study described a process for the analysis of AGVNs, successfully extracted nanoscale AGVNs, analyzed the composition of ginsenosides, and evaluated the anti-inflammatory effect of AGVNs using zebrafish and macrophage models, showing that they have significant anti-inflammatory abilities in vivo and in vitro. This study provides evidence for the development of nanoscale anti-inflammatory substances of natural origin.

## Data Availability

The original contributions presented in the study are included in the article/Supplementary Materials, further inquiries can be directed to the corresponding authors.

## Supplementary Materials

The following supporting information can be downloaded at: https://www.mdpi.com/article/10.3390/molecules29153443/s1, Table S1: Characterization of ginsenosides in AGVNs based on UPLC-Q/TOF-MS/MS.

## Abbreviations

AG	American Ginseng
AGVNs	American-Ginseng-derived vesicle-like nanoparticles
DAPI	2-(4-Amidinophenyl)-6-indolecarbamidine dihydrochloride
DiO	3,3′-dioctadecyloxacarbocyanine perchlorate
DMEM	Dulbecco’s modified Eagle’s medium
ESI	electrospray ion source
IL-6	interleukin-6
IL-10	interleukin-10
LPS	lipopolysaccharide
MS/MS	secondary mass
NO	nitric oxide
OA	oleanolic acid
OPLS-DA	orthogonal partial least squares discriminant analysis
PBS	phosphate-buffered saline
PCA	principal component analysis
PPD	protopanaxadiol
PPT	protopanaxatriol
QC	quality control
TNF-α	tumor necrosis factor-α
UPLC-MS	ultra-performance liquid chromatography coupled with mass spectrometry
VIP	variable importance in projection
VNs	vesicle-like nanoparticles
