# Supplementary material for: Nano-Characterization, Composition Analysis, and Anti-Inflammatory Activity of American-Ginseng-Derived Vesicle-like Nanoparticles
Source: Molecules. 2024 Jul 23;29(15):3443. doi: 10.3390/molecules29153443 (PMC11313632; doi:10.3390/molecules29153443)
Supplement: Supplementary file 1 [file molecules-29-03443-s001.zip › molecules-2970030-supplementary.pdf]

Supplemental information

Table S1. Characterization of ginsenosides in AGVNs based on UPLC-Q/TOF-MS/MS.

No.	m/z	Rt(min)	Error (ppm)	Adducts	Formula	MS/MS	Name	Category
1	1007.5421	1.50	-1.18	M+FA-H	$C_{48}H_{82}O_{19}$	1007.54, 961.54, 637.43,179.05, 161.04	20-Glucoginsenoside Rf	PPT
2	845.4902	2.53	-1.48	M-H, M+FA-H	$C_{42}H_{72}O_{14}$	845.49, 799.48, 637.43, 475.38, 179.06, 161.04,	Ginsenoside Rg1 [a]	PPT
3	977.5318	2.40	-1.93	M-H, M+FA-H	$C_{47}H_{80}O_{18}$	931.52, 637.42, 475.37, 161.05	Notoginsenoside R1	PPT
4	945.5414	2.50	-1.54	M-H	$C_{48}H_{82}O_{18}$	945.54, 799.49, 783.49, 637.43, 475.38, 179.06, 161.04	Ginsenoside Re [a]	PPT
5	955.4900	3.34	-0.80	M-H, M+FA-H	$C_{48}H_{76}O_{19}$	998.51, 955.49, 793.44, 613.37, 455.35,337.08, 279.07	Ginsenoside Ro [a]	OA

No.	m/z	Rt(min)	Error (ppm)	Adducts	Formula	MS/MS	Name	Category
6	1153.6008	3.13	-1.22	M-H, M+FA-H	$C_{54}H_{92}O_{23}$	945.54,783.49,765.48,621.44,603.43,459.38, 323.10, 179.06, 161.05,	Ginsenoside Rb1 [a]	PPD
7	1123.5899	3.21	-1.35	M-H, M+FA-H	$C_{53}H_{90}O_{22}$	1077.59, 945.54, 915.53,799.49,783.49,621.44,459.38	Ginsenoside Rc	PPD
8	1123.5894	3.31	-1.10	M+FA-H	$C_{53}H_{90}O_{22}$	1123.59, 1077.59, 945.54, 915.53,799.49,783.49,621.44,459.38	Ginsenoside Rb2	PPD
9	1195.6109	3.38	-1.49	M-H, M+FA-H	$C_{56}H_{94}O_{24}$	1149.69, 1107.67, 1089.59, 945.54, 927.53, 765.53, 621.48,	Quinquenoside R1	PPD

No.	m/z	Rt(min)	Error (ppm)	Adducts	Formula	MS/MS	Name	Category
10	991.5472	3.53	-1.58	M-H, M+FA-H	$C_{48}H_{82}O_{18}$	603.48, 459.44, 323.12, 221.08, 179.07, 945.54, 783.49, 621.44, 603.43, 459.38, 221.07, 179.06, 161.05	Ginsenoside Rd	PPD
11	1165.5964	3.58	-4.21	M+FA-H	$C_{55}H_{92}O_{23}$	1165.60, 1120.66, 1077.64, 987.61, 879.55, 765.52	Ginsenoside Rs1	PPD
12	845.4902	3.26	-1.97	M-H, M+FA-H	$C_{42}H_{72}O_{14}$	799.48, 653.43, 635.43, 491.38	Pseudoginsenoside F11	PPT
13	811.4841	4.48	-2.19	M-H, M+FA-H	$C_{42}H_{70}O_{12}$	765.47, 619.42, 601.41, 457.36, 205.07, 163.06	Ginsenoside Rg4	PPT
14	829.4943	4.51	-2.70	M-H, M+FA-H	$C_{42}H_{72}O_{13}$	783.49, 621.44, 603.43, 459.38, 179.06	Ginsenoside F2	PPD

No.	m/z	Rt(min)	Error (ppm)	Adducts	Formula	MS/MS	Name	Category
15	829.4949	5.18	-2.13	M-H, M+FA-H	$C_{42}H_{72}O_{13}$	783.49, 621.44, 459.39, 375.29, 161.06	Ginsenoside Rg3	PPD
16	683.4369	3.48	-1.09	M+FA-H	$C_{36}H_{62}O_{9}$	683.43.11, 637.40, 475.36	Ginsenoside Rh1	PPT
17	825.4991	5.23	-1.78	M-H	$C_{44}H_{74}O_{14}$	825.50, 783.49, 765.48, 663.45, 621.44, 459.38, 375.29, 203.06, 161.04	Ginsenoside Rs3	PPD
18	829.4942	3.05	-1.60	M+FA-H	$C_{42}H_{72}O_{13}$	829.49, 783.49, 637.43, 619.17, 475.38	Ginsenoside Rg2	PPT
19	683.4370	3.67	-0.96	M+FA-H	$C_{36}H_{62}O_{9}$	683.44, 637.33, 603.43, 475.38, 179.06	Ginsenoside F1	PPT
20	815.4792	2.85	-0.82	M+FA-H	$C_{41}H_{70}O_{13}$	815.48, 769.47, 637.43, 607.42, 475.38, 391.28, 161.04	Ginsenoside F3	PPT

No.	m/z	Rt(min)	Error (ppm)	Adducts	Formula	MS/MS	Name	Category
21	799.4830	2.91	-2.41	M-H	$C_{42}H_{72}O_{14}$	845.49, 799.49, 653.43, 415.32, 161.05	Ginsenoside Rf	PPT
22	991.5476	3.69	-1.28	M-H, M+FA-H	$C_{48}H_{82}O_{18}$	945.54, 621.44, 603.43, 459.38, 305.08, 263.08, 221.07, 179.06	Gypenoside XVII	PPD
23	1193.5953	3.18	-0.61	M-H	$C_{57}H_{94}O_{26}$	1193.60, 1149.61, 987.55, 927.53, 765.48, 621.44, 603.43	Malonyl-ginsenoside Rb1	PPD
24	1285.6409	2.94	-2.02	M+FA-H	$C_{59}H_{100}O_{27}$	1285.64, 1239.64, 353.11, 149.04	Notoginsenoside Fa	PPD
25	925.4797	3.46	-0.62	M-H, M+FA-H	$C_{47}H_{74}O_{18}$	925.48, 775.43, 613.37, 569.38, 455.35	Pseudoginsenoside RT1	PPD
26	1007.5425	2.92	-0.73	M+FA-H	$C_{48}H_{82}O_{19}$	1007.54, 961.54, 635.42, 179.05, 161.04	Vina-ginsenoside R8	PPD

[a] Retention times and MS/MS were confirmed by reference compounds.
